# Roles of Autotaxin/Autotaxin-Lysophosphatidic Acid Axis in the Initiation and Progression of Liver Cancer

**DOI:** 10.3389/fonc.2022.922945

**Published:** 2022-06-13

**Authors:** Sha She, Qian Zhang, Jinzhi Shi, Fan Yang, Kai Dai

**Affiliations:** Department of Infectious Diseases, Renmin Hospital of Wuhan University, Wuhan, China

**Keywords:** autotaxin, lysophosphatidic acid, tumorigenesis, metastasis, liver cancer

## Abstract

Autotaxin (ATX) is a secreted glycoprotein and catalyzes the hydrolysis of lysophosphatidylcholine to lysophosphatidic acid (LPA), a growth factor-like signaling phospholipid. ATX has been abundantly detected in the culture medium of various cancer cells, tumor tissues, and serum or plasma of cancer patients. Biological actions of ATX are mediated by LPA. The ATX-LPA axis mediates a plethora of activities, such as cell proliferation, survival, migration, angiogenesis, and inflammation, and participates in the regulation of various physiological and pathological processes. In this review, we have summarized the physiological function of ATX and the ATX-LPA axis in liver cancer, analyzed the role of the ATX-LPA axis in tumorigenesis and metastasis, and discussed the therapeutic strategies targeting the ATX-LPA axis, paving the way for new therapeutic developments.

## Introduction

Globally, liver cancer remains a serious health challenge, with an estimated future incidence of > 1 million cases by 2025; moreover, hepatocellular carcinoma (HCC) is the dominant type of liver cancer, accounting for approximately 90% of cases (1). HCC often arises from cirrhosis and is closely related to chronic liver diseases (2). Although surgical resection and liver transplantation may be effective treatments in early liver cancer, most patients are diagnosed at an advanced stage and generally respond poorly to current treatment options. The 5-year overall survival for unresectable HCC is less than 10% (3). According to international oncologic guidelines, sorafenib can be used as first-line treatment in patients with advanced HCC. Unfortunately, it prolongs life expectancy by only a few months, with frequent primary or secondary resistance developing (3, 4). HCC is a complex disease, with multiple molecules and signaling pathways affecting its pathogenesis. More data on the pathogenesis of HCC are required in order to develop new and more effective therapies to improve the survival of HCC patients.

Autotaxin (ATX) is a member of the family of nucleotide pyrophosphatases/phosphodiesterases and is also known as ENPP2. Secreted ATX acts as a lysophospholipase D (LPD), converting extracellular lysophosphatidylcholine (LPC) into lysophosphatidic acid (LPA). Many studies have confirmed that ATX is implicated in various physiological processes and pathological conditions, including cancer (5, 6). For instance, ATX is highly expressed in different kinds of cancers, such as glioblastoma (7), melanoma (8), liver cancer (9), and renal cancer (10). Overexpression of ATX promotes the migration, invasion, and proliferation of cancer cells. Recent studies have shown that most of the biological functions of ATX are attributed to LPA (11). Indeed, ATX has been determined to act as the “gatekeeper” to control LPA signaling through LPA receptors. The ATX-LPA axis is involved in many physiological processes, including embryonic development, angiogenesis, and preadipocyte differentiation (5).

LPA is a small glycerol-phospholipid derived from membrane phospholipids and consists of a fatty acid chain and glycerol backbone. It is involved in a wide range of physiological and pathological responses, including cell proliferation, motility, differentiation, metabolism, excitability, and cell death (12). LPA binds to at least six G protein-coupled receptors (namely LPAR1-6) to stimulate activation of the ATX-LPA signaling pathway and participates in the regulation of tumor occurrence, progression, and metastasis (12, 13). And mounting studies have shown that the ATX-LPA axis can be targeted as an adjuvant for cancer therapy (14).

In recent years, more and more evidence has led to revelations about the key roles of the ATX-LPA axis in HCC pathogenesis. In this review, we summarize the physiological function of ATX and the ATX-LPA axis. Further, we focus on the emerging role of ATX and LPA signaling in HCC and the therapeutic potential of pharmacologically targeting ATX or LPA.

## ATX Structure and Signaling

ATX was originally identified as a secreted phosphatase in conditioned media from A2058 melanoma cells and characterized as an autocrine motility factor (15). ATX and LPA are found at high levels as soluble molecules in the blood, serum, and a wide variety of pathological liquids, such as extracellular space of hepatocytes in chronic hepatitis (16).

ATX consists of a somatomedin-B-like domain, a central phosphodiesterase catalytic domain (PDE), and a C-terminal inactive catalytic nuclease domain (NUC). The architecture of the PDE domain includes the active catalytic site which can bind different LPC and LPA. The NUC maintains the rigidity of the PDE domain. The SMB domain binds to integrins, which is critical for ATX interacting with cell surface integrins, delivering LPA close to its cognate receptors.

**Figure 1:**
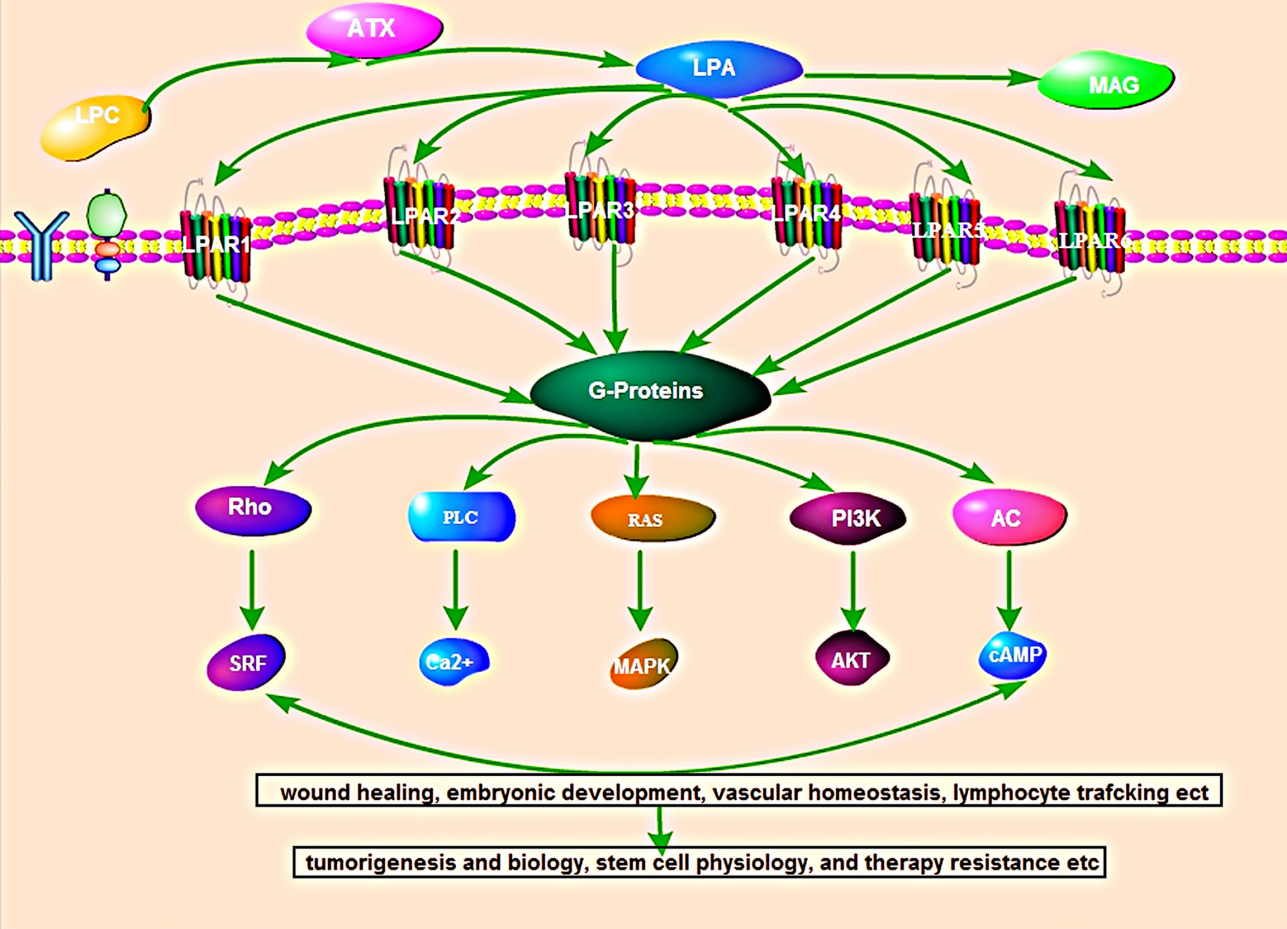
Overview of the autotaxin-lysophosphatidic acid signaling pathway. ATX produces the lipid mediator and GPCR agonist lysophosphatidic acid (LPA) from abundantly available extracellular lysophosphatidylcholine (LPC). LPA signal through its six homologous receptors (LPAR1-6) that is binds to G proteins to activate downstream signaling pathways, including those involving Ras/Raf, RhoA, phosphoinositide 3-kinases, mitogen-activated protein kinases, and protein kinase B/mammalian target of rapamycin. The activated LPA-LPAR pathway participates in wound healing, embryonic development, vascular homeostasis, lymphocyte trafficking, cancer biology, stem cell physiology, and therapy resistance, etc.

Several studies have shown that most of the biological functions of ATX are attributed to signaling by LPA (11). LPA signals through its six homologous receptors (LPAR1-6) that is involved in the regulation of many pathological conditions, including obesity, chronic inflammation, diabetes, neuropathy pain, and cancer (5). Simultaneously, LPAR binds to G proteins to activate downstream signaling pathways, including those involving Ras/Raf, RhoA, phosphoinositide 3-kinases, mitogen-activated protein kinases, and protein kinase B/mammalian target of rapamycin, which play important roles in wound healing, embryonic development, vascular homeostasis, lymphocyte trafficking, cancer biology, stem cell physiology, and therapy resistance, etc. (5) (**Figure 1**).

## Role OF ATX and ATX-LPA Axis in HCC

Increased expression of ATX has been detected in many types of cancers, such as melanoma, breast invasive carcinoma, glioblastoma, lung adenocarcinoma, and ovarian cancer (14). A clinical study showed that serum ATX levels were significantly elevated in breast cancer patients. Further research found that overexpression of ATX promotes the migration and invasion of MDA-B02 cell (17). In ovarian cancer, ATX regulates the activity of ovarian cancer stem cells through an LPA-mediated autocrine mechanism (18). And ATX-LPA axis has important effects on both tumorigenesis and cancer cell invasion. Studies have shown that the ATX-LPA axis could have direct oncogenic effects on normal cells. Liu et al (19) found that forced expression of ATX or one of the three LPA receptors in the mammary glands of transgenic mice can induce the spontaneous development of breast cancer. Further studies have shown that the ATX-LPA axis also enhances tumor aggressiveness. ATX-LPA axis plays a crucial role in cancer cell proliferation and growth, motility, invasion, and tumor tissue angiogenesis (18–21). As the important role of ATX/LPA in cancer, this review focused on the role and possible mechanism of ATX/LPA in HCC.

### Aberrant Expression of ATX and LPA in HCC

Recent studies have shown that ATX and LPA are highly expressed in HCC. The serum ATX activity and plasma LPA levels are significantly increased in patients with HCC *vs* normal patients. Also, ATX transcripts, along with LPA and LPA receptor protein, are significantly expressed in HCC tissues compared with normal tissues (22).

Wu’s study demonstrated that ATX protein was over-expressed in human HCC tissues and human hepatoma cell lines compared with that in normal controls for the first time (23). Memet et al. (24) further confirmed that high ATX expression in HCC was detected in patients with histological grade II and III. Further, patients with elevated ATX expression levels were found to possess an 8-fold higher risk of death. But, controversy exists about ATX expression in HCC. Studies have shown that the increase in serum ATX levels in HCC patients may not be caused by abundant ATX production in HCC tissues but by inflammation and/or fibrosis in the background livers (25). ATX levels positively correlated with liver fibrosis stage and served as a predictor of liver disease severity and overall survival. Similarly, ATX was found to be significantly elevated in hepatitis-related HCC tissues (26). The collective findings from these studies suggest that ATX upregulation in HCC requires the presence of an inflammatory and fibrotic component that exists in liver. The latest research shows that hepatitis virus infection and/or fibrosis increases hepatocellular ATX expression that establishes a paracrine ATX-LPA signaling environment leading to HCC pathogenesis (26). On the other hand, highly expressed ATX can amplify pro-inflammatory and pro-fibrosis signaling pathways to induce HCC (27).

Indeed, Mazzocca et al. (28) found that LPA serum levels were higher in HCC patients than in healthy controls or liver cirrhosis patients. Among HCC patients, LPA serum levels were higher in those with metastasis compared to those without. Moreover, patients with higher serum levels of LPA also have larger HCC tumors and shorter survival compared with those with lower LPA serum concentrations (28). As mentioned above, adipose tissue expresses ATX abundantly. The ATX-LPA signaling axis could be proposed as a possible molecular pathogenic link between metabolic disorders and HCC (28). In general, ATX-LPA is highly expressed in HCC and is related to the occurrence and progression of HCC.

### ATX-LPA Axis in HCC Tumorigenesis and Metastasis

As a motor factor, the ATX-LPA axis plays a direct role in liver pathophysiology. ATX-LPA axis is involved in tumor-promoting inflammation. It reported that ATX-LPA interacts with the tumor necrosis factor-nuclear factor-*κ*B axis to regulate inflammatory liver tumorigenesis (23). ATX-LPA activation can disrupt lipid homeostasis, increase hepatic stellate cell activation, amplify fibrillation signals, and ultimately lead to the occurrence of HCC (27). Moreover, ATX-LPA axis plays an important role in regulating the biological behavior of HCC cells, thus promoting tumor progression (28). Zhang et al. (29) provided the first evidence that the ATX-LPA axis induces the expression of osteopontin to promote the migration of SMMC7721 cells. Other studies showed that ATX-LPA increases HCC cell invasion, proliferation and motility (24, 26). In addition, ATX-LPA axis induced epithelial-mesenchymal transition phenotype of HCC and HCC angiogenesis to accelerate the metastasis of HCC (23, 28). All these studies indicate that ATX/LPA axis plays an important role in the occurrence and progression of HCC.

**Figure 2:**
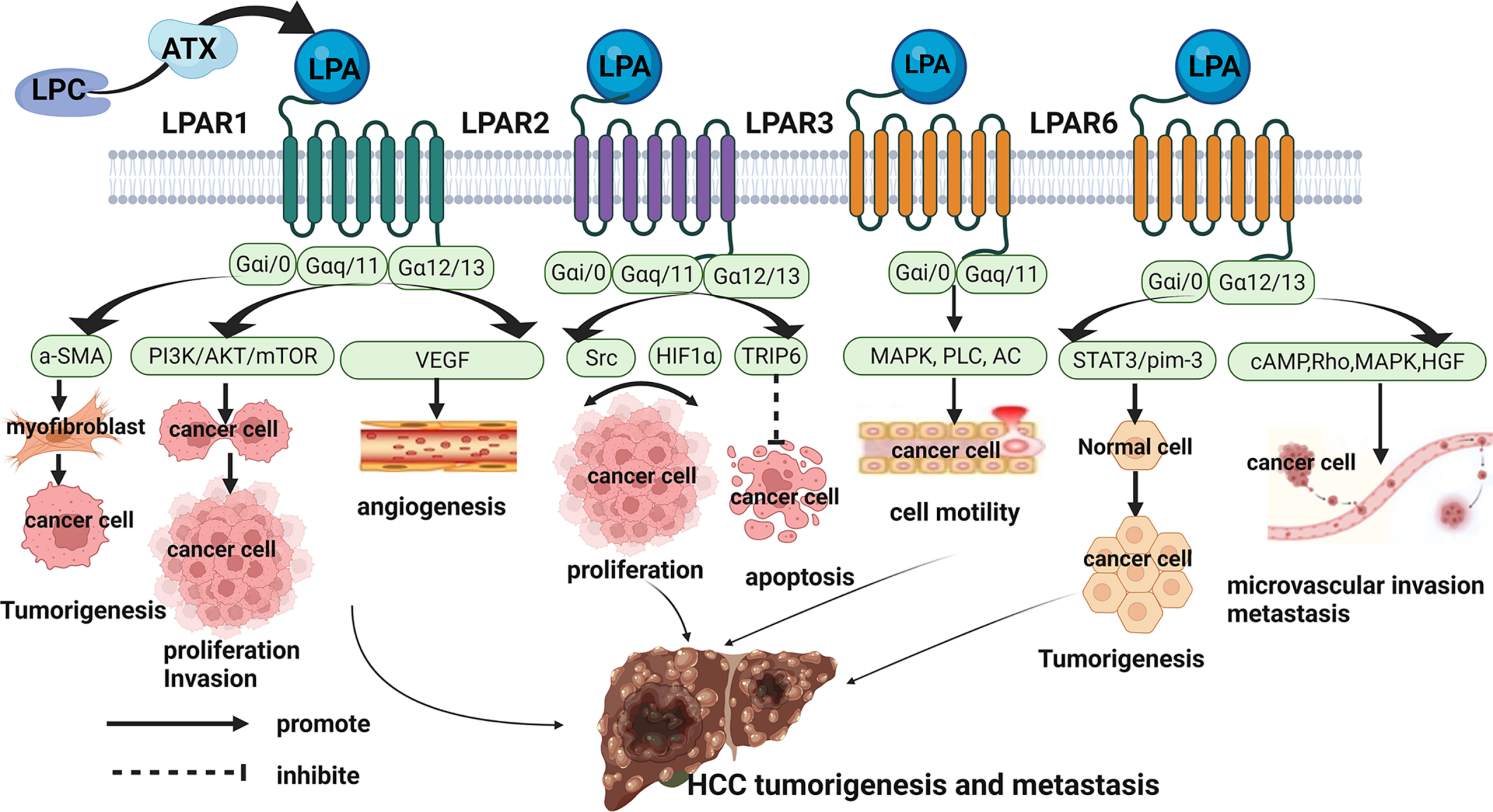
Role and Mechanisms of ATX/LPA in hepatocellular carcinoma. LPAR1 promoted the occurrence, proliferation, migration and angiogenesis of HCC by coupling with Gαi/0, Gαq/11, and Gα12/13, and activating downstream a-SMA, PI3K/AKT/mTOR, VEGF pathways. LPAR2 may regulate the proliferation of HCC cells through Src signaling pathway and HIF1α pathway. On the other hand, LPAR2 may interact with TRIP to inhibit apoptosis of HCC cells. LPAR3 promoted tumor cell motility in HCC through coupling to Gαi/0 and Gα11/q to mediate downstream activation of MAPK, PLC, and inactivation of AC. LPAR6 supported the tumorigenicity of HCC through a STAT3/pim-3-dependent mechanism. And LPAR6 increased microvascular invasion in HCC through mediating cAMP reduction, Rho-dependent morphological changes, MAPK and HGF activation.

Hence, the underlying mechanisms of ATX-LPA axis activation in HCC are a topic of concern. As previously mentioned, ATX-LPA axis is involved in the regulation of many pathological conditions through its six homologous G protein-coupled receptors (LPAR1-6). According to the homology, the LPA receptor can be subdivided two families: the endothelial differentiation gene (EDG) family and the non-EDG family. LPA1, LPA2 and LPA3 belong to the Endothelial Differentiation Gene (EDG) family, whereas LPA4, LPA5, and LPA6 represent a sub-cluster of the Purinergic GPCR family. LPA receptors use at least two Gα subunits (Gα12/13, Gα Q/11, Gα I/O, and GαS) to signal, thereby activating different downstream pathways. In recent years, there is a lot of evidence that LPA-LPAR axis plays an indispensable part in the occurrence and development of cancer. This review further explores the role and mechanism of LPAR in the occurrence and progression of HCC, and provides a new direction for the mechanism of ATX-LPA axis activation in HCC (**Figure 2**).

LPAR1, also known as EDG2, is widely expressed in various tissues and organs of human body. It couples with and activates 3 types of G protein, Gαi/0, Gαq/11, and Gα12/13, to mediate cellular processes, cytoskeletal changes, Ca2+ mobilization, immune function, and myelination by initiating downstream signaling including PI3K/AKT, Rho, MAPK, and PLC. Numerous studies have documented a critical role for LPAR1 in enhancing tumor motility and metastasis (13). In HCC, LPAR1 expression was significantly increased (25). LPAR1 promoted hepatocarcinogenesis by mediating the recruitment and activation of fibroblasts (30). Moreover, LPAR1 was found to promote viability and proliferation of HCC cell through PI3K/AKT/mTOR signaling (31). Another study showed that ATX-LPA increases HCC cell invasion and subsequent production of matrix metallopeptidase 9 by a LPAR-dependent mechanism (32). Vascular endothelial growth factor may stimulate LPA production by inducing ATX expression and increase LPAR1-mediated intracellular signaling to promote HCC angiogenesis (33). Taken together, these data confirmed the important role of LPAR1 in the occurrence and development of HCC.

LPAR2 (EDG4) is the same as LPAR1, is also coupled with Gαi/o, Gαq/11 and Gα12/13 in the heterotrimeric G protein family. Its activation is associated with tumor cell survival and migration through Ras, MAPK, phosphatidylinositol 3-kinase (PI3K), Rac, PLC, diacylglycerol (DG) and Rho and other downstream molecules transmit signals. By analyzing liver cancer tissues and adjacent tissues of 58 HCC patients, Enooku et al. found that the high LPA2 mRNA levels in HCC correlated with a poorer differentiation of HCC and were a risk factor for recurrence when combined with serum ATX levels (34). However, the role and mechanism of LPAR2 in the occurrence and progression of HCC is not clear. In other tumors, such as colon cancer and breast cancer ect, LPAR2 promotes the migration, invasion, and proliferation of tumor cells through LPAR2-Gi-Src-EGFR-ERK signaling and HIF1α-LPA-LPAR2 Axis (35, 36). On the other hand, LPAR2 can also promote migration protect cancer cells against apoptotic stress after irradiation and chemotherapy through the focal adhesion molecule TRIP6 (37). These studies provide direction to explore the role and mechanism of LPAR2 in HCC in the future.

LPAR3 (EDG7) has 52% and 48% homology with LPA1 and LPA2, respectively, and couples to G proteins, Gαi/0 and Gα11/q to mediate downstream activation of MAPK, PLC, and inactivation of AC. It is the predominant receptor subtype in different kinds of cancers including HCC, and promotes tumor cell motility and invasiveness. In a study by Zuckerman and coworkers, LPAR3 was high expressions in HCC tissues, and it may enhance HCC cells migration *via* the LPAR3-Gi-ERK/MAPK pathway (38). Okabe et al. found LPAR3 expression in rat hepatic RH7777 cancer cells contributes to elevate cell motility and invasive capability *via* the β-catenin pathway (39). They also demonstrated that cells survival of LPAR3-expressing was higher than control cells upon treatment with cisplatin or doxorubicin through multidrug-resistance-related up-regulation of genes (39).

LPAR6 (P2Y5) is the most recently identified LPA receptor, it binds Gαi/0 and Gα12/13 to mediate cAMP reduction, Rho-dependent morphological changes, Ca2+ mobilization and MAPK activation. The first demonstrated function of LPAR6 was to regulate human hair growth, and recent studies have found that LPAR6 is involved in tumorigenesis and progression (36). In HCC, LPAR6 expression correlated with poorer survival and increased microvascular invasion (34). And LPAR6 supported the tumorigenicity of HCC through a STAT3/pim-3-dependent mechanism (40). Zheng’s study indicated that LPAR6 promotes HCC proliferation *via* the NCOA3-LPAR6-HGF signaling cascade (41). Research on the role and mechanism of LPAR6 in HCC is still relatively rare, which may be a future research direction.

In contrast to LPAR1-3 and LPAR6, LPAR4 and LPAR5 negatively affected cancer cell proliferation and motility (42). There have no articles about LPAR4 and LPAR5 in HCC, which may be a future research direction.

## Therapeutic Potential of ATX and the ATX-LPA Axis

As the ATX-LPA axis plays key roles in promoting tumor migration, metastasis, invasion, and angiogenesis, blocking the ATX-LPA signaling pathway would be an effective and important method for the treatment of cancers. Several molecules have been proposed as candidates with promising results, including Ki16452 (a LPAR1 and LPAR3 antagonist); ONO-8430506(an ATX inhibitors); GLPG1690 (an ATX inhibitors) and LPAR6 antagonists.

Ki16425 was synthesized by the OHTA team, selectively inhibits LPA receptor-mediated actions including DNA synthesis and cell migration (43). Further research found that Ki16425 preferentially inhibited LPA1- and LPA3-mediated actions. In HCC, a study reported that the Ki16452 may significantly reduce LPA-related HCC cell invasion (28).

ONO-8430506 was attracted attention as a novel ATX inhibitor. Studies found that ONO-8430506 had a long-term effect of decreasing the activity of ATX activity and LPA production (44). Therefore, it effectively breaks the inflammatory cycle by reducing the concentration of inflammatory cytokines in the tumor microenvironment, resulting in decreased tumor growth (44). Another study found that ONO-8430506 enhanced the antitumor effect of paclitaxel in a breast cancer model (45).

GLPG1690 is a first-in-class inhibitor of ATX and sought to interfere with the treatment of fibro-proliferative diseases (46). As the first ATX clinical candidate, GLPG1690 is currently being evaluated in phase III clinical trials in Pulmonary fibrosis (47). A recent study found that GLPG1690 suppressed TSC2-loss associated oncogenicity *in vitro* and *in vivo* through inhibiting AKT and ERK1/2 signaling (48). Another study found that GLPG1690 increased the efficacy of radiotherapy and chemotherapy in a mouse model of breast cancer (49). Due to its promising drug-ability, a substantial amount of efforts has been invested in developing analogs of GLPG1690 by scientists in pursuit of potent ATX inhibitors.

As LPA receptor 6 (LPAR6) is essential for supporting the tumorigenicity of HCC, a substantial amount of efforts has been invested in developing LPAR6 inhibitors. Team of Mazzocca identified two promising candidates, namely 4-methylene-2-octyl-5-oxotetra-hydrofuran-3-carboxylic acid (C75) and 9-xanthenylacetic acid (XAA) (50). Further research confirmed that both C75 and XAA inhibited HCC cell growth by causing a G1-phase arrest (50, 51).

## Conclusion

Evidence demonstrates that alterations of ATX-LPA signaling promote HCC development and progression *via* different mechanisms. It suggests that targeted inhibition of the ATX-LPA pathway may be a new therapeutic strategy for inhibiting HCC metastasis and drug resistance. The LPAR inhibitors discovered in the latest research inhibit the growth of HCC by affecting the function of mitochondria of HCC cells (50). These inhibitors have the specific potential to be converted into effective molecules with no side effects for HCC treatment. A new study finds that the ATX-LPA signaling axis creates a T cell-excluding, pro-tumorigenic microenvironment that is amenable to therapeutic intervention. According to the latest guidelines, the combination of targeted drugs and immunotherapy is the best recommended treatment for advanced HCC. Based on this, it is likely that targeting ATX-LPA signaling will develop into an important part of a comprehensive strategy to combat advanced HCC and bring a brighter future to liver cancer patients. It still needs to be supported by more studies on ATX-LPA axis in HCC in the future.

## Author Contributions

SS and QZ performed the majority of the writing and prepared the figures; JS carried out the literature review for data collection and coordinated the writing of the paper; KD and FY designed the study and revised the manuscript. All authors read and approved the final manuscript.

## Funding

This work supported by the National Natural Science Foundation of China, No. 81972673.

## Conflict of Interest

The authors declare that the research was conducted in the absence of any commercial or financial relationships that could be construed as a potential conflict of interest.

## Publisher’s Note

All claims expressed in this article are solely those of the authors and do not necessarily represent those of their affiliated organizations, or those of the publisher, the editors and the reviewers. Any product that may be evaluated in this article, or claim that may be made by its manufacturer, is not guaranteed or endorsed by the publisher.
